# PI3K/Akt/mTOR signaling pathway mediates energy metabolic reprogramming and regulates mitochondrial homeostasis in host cells exposed to *Toxoplasma gondii*

**DOI:** 10.1128/spectrum.01385-25

**Published:** 2025-12-22

**Authors:** Kangzhi Xu, Shifan Zhu, Jing Ma, Mingyue Zu, Jin Yang, Fan Xu, Linwei Dai, Dandan Liu, Yanhong Wang, Xinjun Zhang, Siyang Huang, Jinjun Xu, Zhiming Pan, Jianping Tao, Zhaofeng Hou

**Affiliations:** 1 College of Veterinary Medicine, Yangzhou University, Yangzhou, China; 2 Jiangsu Co-innovation Center for Prevention and Control of Important Animal Infectious Diseases and Zoonoses, Yangzhou University, Yangzhou, China; 3 Jiangsu Key Laboratory of Zoonosis, Yangzhou University, Yangzhou, China; 4 Northern Jiangsu People’s Hospital Affiliated to Yangzhou University 38043, Yangzhou, China; Universidade de Brasilia, Brasilia, Brazil

**Keywords:** *Toxoplasma gondii*, energy metabolic reprogramming, mitochondrial dynamics, PI3K/Akt/mTOR, LY294002

## Abstract

*Toxoplasma gondii* (*T. gondii*) relies on host cells for energy and nutrition. Our previous studies showed that *T. gondii* regulates host cell apoptosis via the mitochondrial pathway, highlighting the essential role of mitochondria in its parasitism. In this study, *T. gondii* infection was found to significantly affect mitochondrial morphology and dynamic homeostasis in porcine kidney-15 (PK-15) cells, characterized by aggregated, swollen, fragmented, and oval-shaped mitochondria with disappearing cristae, accompanied by increased fusion and decreased fission. Additionally, the energy metabolic reprogramming of PK-15 cells exposed to *T. gondii* was affirmed from the perspectives of glucose consumption; changes in NAD^+^/NADH, lactate, pyruvate, and ATP production; and expressions of proteins related to glycolysis and oxidative phosphorylation (OXPHOS). *T. gondii*-induced mitochondrial damage impaired the OXPHOS process; however, the glycolysis level was significantly increased. Mechanistically, we demonstrated that activation of phosphatidylinositol 3-kinase (PI3K)/protein kinase B (Akt)/mammalian target of rapamycin (mTOR) signaling pathway played a critical role in energy metabolic reprogramming and mitochondrial damage induced by *T. gondii*, and this effect could be attenuated by LY294002 (a PI3K inhibitor), which significantly reduced intracellular proliferation of *T. gondii* through inhibiting PI3K/Akt/mTOR signaling pathway. These findings highlight the PI3K/Akt/mTOR pathway as a key mediator of *T. gondii*-induced cellular metabolic reprogramming and mitochondrial dysfunction; however, its potential as a therapeutic target remains to be validated *in vivo*.

**IMPORTANCE:**

*Toxoplasma gondii*, a globally distributed obligate intracellular protozoan parasite, poses severe health risks to immunocompromised individuals and pregnant women, causing miscarriage and fetal abnormalities. Current therapies suffer from high toxicity and limited targets, with unclear mechanisms underlying host-parasite interactions. This study reveals a novel parasitic strategy: *T. gondii* hijacks host mitochondrial dynamics and energy metabolism. Infection disrupts mitochondrial morphology and suppresses oxidative phosphorylation while activating the PI3K/Akt/mammalian target of rapamycin (mTOR) pathway to drive metabolic reprogramming, enhancing glycolysis to meet energy demands. Critically, inhibiting PI3K/Akt/mTOR with LY294002 reduces intracellular parasite proliferation, validating this pathway as a therapeutic target. Conventional antiparasitic drugs targeting the parasite directly face resistance challenges. By focusing on host metabolic regulation via PI3K/Akt/mTOR, this work advances understanding of parasitism and proposes host-directed therapies to disrupt parasite proliferation by modulating the metabolic microenvironment, highlighting its therapeutic potential against toxoplasmosis.

## INTRODUCTION

*Toxoplasma gondii* is an obligate intracellular parasitic protozoan that can cause zoonotic diseases with a widespread geographic distribution (1). Toxoplasmosis is linked to a range of behavioral alterations, and a poor prognosis usually occurs in newborns and immunosuppressed individuals exposed to the infection (2). Human toxoplasmosis outbreaks are frequently linked to the consumption of raw or undercooked meat, particularly from infected pigs or wild boars (3, 4), accounting for 12% of the global disease burden (5). Therefore, controlling toxoplasmosis in pigs is crucial for maintaining public health, food safety, and the sustainable development of the livestock industry (2). However, current approaches to addressing toxoplasmosis in both humans and animals remain considerably inadequate (6).

As an intracellular parasite, *T. gondii* replicates within host cells during both its tachyzoite and bradyzoite (cyst) stages by extracting nutrients from the host. It can globally reprogram key host metabolic pathways, such as glycolysis, lipid metabolism, and cholesterol metabolism, to fulfill its growth and reproductive needs (7, 8). Conversely, host cells can also control parasite growth by restricting the metabolism of certain nutrients (9, 10). Therefore, the interaction between *T. gondii* and host cell metabolism plays a significant role in the development and pathogenesis of this parasite.

Mitochondria are responsible for energy metabolism in cells and serve as the main source of energy for normal cells by producing ATP through oxidative phosphorylation (OXPHOS). It has been shown that mitochondrial function is essential for the division and proliferation of intracellular parasitic tachyzoites of *T. gondii* (11). Proteomic and transcriptomic analyses of host cells following *T. gondii* infection revealed that one-third of the regulated proteins were localized to the mitochondria (7), and mitochondrial-dominated metabolic pathways were disrupted (12, 13). *T. gondii*-infected host cells exhibit metabolic behaviors similar to those of tumor cells, maintaining glycolytic metabolism even under aerobic conditions. This shift results in changes in metabolic intermediates and products, a phenomenon known as metabolic reprogramming, often referred to as the “Warburg effect” (14, 15). By sustaining high levels of glycolytic intermediates and elevated lactate production, *T. gondii* enhances biosynthetic activity (16), creating an environment that promotes the continued development of tachyzoites instead of transitioning to bradyzoites (17). Therefore, metabolic reprogramming in host cells significantly influences the growth rate and morphology of *T. gondii* (18).

Prior studies have revealed that differentially expressed genes in host cells induced by *T. gondii* invasion are significantly enriched in the PI3K/Akt/mammalian target of rapamycin (mTOR) signaling pathway (19). This pathway is considered a crucial mediator in various cellular physiological processes, such as inflammation, the cell cycle, aging, and apoptosis, and is currently a key research focus in the study of metabolism (20, 21). When PI3K is activated, it catalyzes the phosphorylation of phosphatidylinositol 4,5-bisphosphate to form phosphatidylinositol 3,4,5-trisphosphate (PIP3). PIP3 then recruits 3-phosphoinositide-dependent protein kinase-1 (PDK1) and Akt proteins to the plasma membrane, leading to the phosphorylation of Akt by PDK1 (22). Akt activation enhances glucose uptake in cancer cells by regulating glucose transporter-1 (GLUT1) (23). In addition to increasing glucose uptake, Akt also regulates several glycolytic enzymes, such as hexokinase 2 (HK2) and phosphofructokinase (PFK), through both post-translational and transcriptional mechanisms (24). PI3K-dependent Akt activation leads to increased PFK2 phosphorylation and the production of fructose-2,6-bisphosphate, which promotes PFK1 activation. This enhanced PFK1 activation and GLUT1 expression, driven by the PI3K/Akt pathway, promote the Warburg effect, cell proliferation, and tumorigenesis (25). Activation of mTORC1 elevates hypoxia-inducible factor-1α levels, inducing aerobic glycolysis in tumor cells (26). These findings underscore the critical role of the PI3K/Akt/mTOR pathway in regulating metabolism.

In previous studies, we have found that *T. gondii* mediates apoptosis of host cells and metabolic disturbances through the mitochondrial pathway. However, the molecular processes by which *T. gondii* induces mitochondrial dysfunction and reprograms cellular energy metabolism remain unclear. High-throughput sequencing results from *in vivo* and *in vitro* infection models have shown that during *T. gondii* infection, cellular energy metabolism is synergistic with the expressions of PI3K/Akt/mTOR signaling pathway, which is highly likely to play an important role in *T. gondii*-induced mitochondrial dysfunction and cellular energy metabolism reprogramming. The findings of this study will contribute to a better understanding of how *T. gondii* regulates host cell metabolism and provide a theoretical foundation for further developing the key targets to limit the intracellular parasitism and proliferation of *T. gondii*.

## MATERIALS AND METHODS

### Parasites and cells

The human foreskin fibroblast (HFF) and porcine kidney-15 (PK-15) cell lines, originally sourced from ATCC, were purchased from Shanghai Zhong Qiao Xin Zhou Biotechnology Co., Ltd. (Shanghai, China) and cultured in Nunc Easyflasks (Thermo Scientific, Waltham, MA, USA). The cells were maintained in Dulbecco’s Modified Eagle Medium (DMEM; Thermo Scientific) supplemented with 10% fetal bovine serum, 100 U/mL penicillin, and 10 mg/mL streptomycin at 37°C in a 5% CO_2_ atmosphere. Cells were used between passages 3 and 16 in experiments.

The *T. gondii* RH strain expressing tomato red fluorescent protein (RH-RFP), constructed under the tubulin promoter as described by Striepen et al. (27), was kindly provided by Siyang Huang, College of Veterinary Medicine, Yangzhou University. RH-RFP was cultured in monolayers of HFF cells. To harvest tachyzoites, heavily infected cells were scraped and passed through a 27-gage needle 3–5 times. Cell debris was removed using a 3 µm membrane filter (Whatman, Maidstone, UK), and tachyzoites were counted using a hemocytometer for further experiments.

### Antibodies and reagents

Anti-Bcl-2 Associated X-protein (BAX; #A19684), anti-B-cell lymphoma 2 (BCL-2; #A19693), anti-PI3K (#A22730), anti-phospho-PI3K-P85α (#AP0854), anti-Akt (#A22738), anti-phospho-Akt-S473 (#AP1208), anti-mTOR (#A2445), anti-phospho-mTOR-S2448 (#AP0115), anti-GLUT1 (#A11727), anti-6-Phosphofructo-2-Kinase (PFKFB2; #A9311), anti-HK2 (#A22319), anti-PDK1 (#A8930), anti-Lactate Dehydrogenase A (LDHA; #A0861), anti-Succinate Dehydrogenase Complex Subunit B (SDHB; #A23832), anti-Cytochrome c Oxidase Subunit 5B (COX5B; #A23762), anti-ATP Synthase Membrane Subunit C Locus 1 (ATP5G1; #A20960), anti-α-Tubulin (#AC012), HRP-conjugated Goat anti-Rabbit IgG (#AS014), and HRP-conjugated Goat anti-Mouse IgG (#AS003) were purchased from ABclonal Technology (Wuhan, Hubei, China). Anti-Pyruvate Kinase M2 (PKM2; #D78A4) and anti-Cleaved-Caspase 3 (Cl-Cas3; #9661) were obtained from Cell Signaling Technology (Danvers, MA, USA). Anti-NADH: Ubiquinone Oxidoreductase Subunit B8 (NDUFB8; #67690-1-Ig) and anti-Ubiquinol-Cytochrome C Reductase Core Protein 1 (UQCRC1; #67888-1-Ig) were sourced from Proteintech Technology (Wuhan, Hubei, China). Anti-Mitofusin 1 (MFN1; #TD7543), anti-Mitochondrial Fission 1 Protein (FIS1; #TD12005), anti-Optic Atrophy 1 (OPA1; #TD8587), and anti-NDUFB9 (#TD4208) were provided by Abmart Technology (Shanghai, China). RIPA Lysis Buffer (#WB3100) and Protease and Phosphatase Inhibitor Cocktail (#P002) were purchased from New Cell and Molecular Biotech (Suzhou, Jiangsu, China). Mito-Tracker Green (#C1048), Enhanced NAD^+^/NADH Assay Kit with WST-8 (#S0176S), Enhanced Mitochondrial Membrane Potential Assay Kit with JC-1, and Enhanced ATP Assay Kit (#S0027) were obtained from Beyotime (Shanghai, China). FastPure Cell/Tissue Total RNA Isolation Kit (#RC112), HiScript III All-in-one RT SuperMix Perfect for quantitative PCR (qPCR; #R333), and AceQ Universal SYBR qRT-PCR Master Mix Kits (#Q511) were acquired from Vazyme (Nanjing, Jiangsu, China). LY294002 (#HY-10108) was purchased from MedChem Express (Monmouth Junction, NJ, USA). Cell Counting Kit-8 was obtained from Dojindo Laboratories (Kumamoto, Japan). Glucose Assay Kit (#A154-1-1), Pyruvate Assay Kit (#A081-1-1), and Lactic Acid Assay Kit (#A019-2-1) were purchased from Nanjing Jiancheng Bioengineering Institute (Nanjing, Jiangsu, China).

### Flow cytometry

The mitochondrial membrane potential (ΔΨm) levels of PK-15 cells were determined using the Enhanced Mitochondrial Membrane Potential Assay Kit with JC-1, following the manufacturer’s instructions. Briefly, PK-15 cells were seeded at a density of 3 × 10⁵ cells per well in a six-well plate. After 12 h, when the cell density reached 60%–80%, one well of cells was collected and counted. Fresh *T. gondii* RH-RFP strain tachyzoites were simultaneously purified from HFF cells and counted. Tachyzoites were added to the six-well plates at a multiplicity of infection (MOI) of 5:1 and cultured for 0, 12, 24, and 36 h. After the desired infection time, cells were harvested, resuspended in 500 μL of culture medium, and incubated with 500 μL of JC-1 staining solution at 37°C for 20 min. After incubation, cells were collected by centrifugation at 600 × *g* and 4°C, followed by two washes with JC-1 staining buffer. The ΔΨm levels were analyzed using a CytoFLEX Flow Cytometer (Beckman, Shanghai, China).

### Transmission electron microscopy

Cells from different groups were collected using a cell scraper, fixed in 2.5% glutaraldehyde, and washed three times with 0.1 M phosphate-buffered saline (PBS). Subsequently, the cells were fixed again with 1% osmium tetroxide for 1–2 h, followed by three additional washes with PBS. The samples were dehydrated using a graded series of ethanol solutions (30%–100%) and then treated with pure acetone. They were embedded in a graded mixture of embedding agent and acetone, followed by pure embedding agent overnight. The embedded samples were heated at 70°C overnight. Ultrathin sections (70–90 nm) were cut, stained with lead citrate and uranyl acetate, and observed under a transmission electron microscope (TEM; HITACHI H-7650, Tokyo, Japan). The mitochondrial outline was manually traced in TEM images using ImageJ, and the enclosed area was measured (28).

### Glucose consumption, pyruvate, lactate, NAD^+^/NADH, and ATP production assays

PK-15 cells were seeded at a density of 3 × 10⁵ cells per well in six-well plates. After 12 h, when cell confluence reached 60%–80%, one well of cells was collected and counted. Fresh tachyzoites were added to the six-well plates at an MOI of 5:1 and cultured for 0, 12, 24, and 36 h. Following the designated infection times, the cell culture supernatants were collected to measure glucose, pyruvate, and lactate levels, while the cell pellets were harvested for NAD^+^/NADH and ATP measurements using corresponding commercial kits, following the manufacturer’s instructions. Glucose and pyruvate concentrations were determined by measuring the optical density (OD) at 505 nm, and lactate concentration was measured at 530 nm. Glucose consumption for each group was calculated by subtracting the glucose content in the cell culture supernatant from that in the cell-free medium. Intracellular NAD^+^/NADH and ATP levels were determined by measuring the OD at 450 nm and bioluminescence using a Microplate Absorbance Reader (Bio-Rad, Hercules, CA, USA) and a Tecan Infinite 200 PRO microplate reader (Tecan Group Ltd., Männedorf, Switzerland), respectively. For metabolite analysis, all experiments were performed at least three times with more than three replicates per sample.

### Quantitative real-time PCR

For total RNA extraction, 3 × 10⁵ cells were seeded in six-well tissue culture-coated plates in 2 mL of medium, with or without LY294002. Fresh tachyzoites were added to a final amount of 1.5 × 10^6^ with an MOI of 5 at 12 h post-seeding. After the desired infection time, the wells were washed twice with PBS, and 500 μL RNA-easy isolation reagent was added into per well. The total RNA of cells was extracted using the FastPure Cell/Tissue Total RNA Isolation Kit following the instructions and quantified by the ratio of absorbance (260 nm/280 nm) using a Nanodrop spectrophotometer (Thermo Scientific). Reverse transcription of 1 μg RNA was performed using the HiScript III All-in-one RT SuperMix Perfect for qPCR Kits with the manufacturer’s instructions. qPCR assays were performed by 7500 Fast Real-Time PCR System (Applied Biosystem, CA, USA) using the AceQ Universal SYBR qPCR Master Mix Kits with the optimized primers. Based on the 2^−ΔΔCt^ method (29) method, the relative expression levels of the target genes (*PKM2*, *GLUT1*, *PDK1*, *PFKFB2*, *HK2*, *LDHA*, *NDUFB8*, *NDUFB9*, *SDHB*, *UQCRC1*, *COX5B,* and *ATP5G1*) were quantified and normalized using the internal control gene (*α-Tubulin*). The primer sequences are presented in Table 1.

**TABLE 1:** Primer sequences used in the experiments

Name	Sequence (5′–3′)
qPCR-MFN1-F	CTAACAATCCAGCAACACCAGATAATG
qPCR-MFN1-R	CCAAATCACTCCTCCAACAACAATG
qPCR-FIS1-F	GCAGACAGAGCCACAGAACAAC
qPCR-FIS1-R	AGTCCAATGAGTCCAGCCAGTC
qPCR-OPA1-F	AAGTTCTTGATGTTCTGTCTGATTATGATG
qPCR-OPA1-R	GCACTCTGATCTCCAACCACAAC
qPCR-BAX-F	GGAGATGAACTGGACAGTAAC
qPCR-BAX-R	GCCGTCAGCAAACATTTC
qPCR-BCL-2-F	GCACCTGACTCCCTTCA
qPCR-BCL-2-R	ACTCAAAGAAGGCCACAATC
qPCR-PKM2-F	GGCTCGTGGTGATCTAGGCATTG
qPCR-PKM2-R	GTGGCACAGATGACAGGCTTCC
qPCR-GLUT1-F	ACGGTGCTCCTGGTCCTGTTC
qPCR-GLUT1-R	CTCGGGTGTCTTGTCGCTTTGG
qPCR-PDK1-F	CACCAGGACAGCCAATACAAGTGG
qPCR-PDK1-R	TGCTCCATCGTTGCTCTCATTGC
qPCR-PFKFB2-F	CGGCGTGAAGCGGTCAAGTC
qPCR-PFKFB2-R	CACTGCGATCTGCCCGTTGTC
qPCR-HK2-F	TCAAGGAGAACAAGGGCGAGGAG
qPCR-HK2-R	TCAGAGCGGAGGAAGCGGATG
qPCR-LDHA-F	CAGGTGGTGGACAGTGCTTATGAG
qPCR-LDHA-R	GGATGCACCCGCCTAAGATTCTTC
qPCR-NDUFB8-F	ACTACGAGCCTTACCCAGATGATG
qPCR-NDUFB8-R	GTCTAGGTCCCAGTGTATCGGTTC
qPCR-NDUFB9-F	TCAGCACCCTCAGCCATACATC
qPCR-NDUFB9-R	GTCATCTAAGCACCACTCAGGAAC
qPCR-SDHB-F	TGAATAACTGTGGTCCTATGGTGTTG
qPCR-SDHB-R	AGTGTTGCCTCCGTTGATGTTC
qPCR-UQCRC1-F	GCCCCTCGAATGGTGCTTGC
qPCR-UQCRC1-R	TCCCAGAGAGGCTGCTGAAGTG
qPCR-COX5B-F	CAAGCGGATAGTGGGCTGCATC
qPCR-COX5B-R	GCTGGGTCTCGCCTTTGTGC
qPCR-ATP5G1-F	TGCCAGGAACCCATCTCTGAAG
qPCR-ATP5G1-R	AGAGGATAAGGAAGGCGACCATC
qPCR-α-Tubulin-F	CCACAGTCCCACAGGTGAACAAC
qPCR-α-Tubulin-R	GCTCTCTTGGGCGGCAGTTTC

### Western blotting

Total proteins were extracted from cells using RIPA lysis buffer supplemented with a protease and phosphatase inhibitor cocktail. Equal amounts of protein (50 μg) were separated by sodium dodecyl sulfate–polyacrylamide gel electrophoresis and transferred to polyvinylidene fluoride membranes. The membranes were incubated overnight at 4°C with primary antibodies, washed five times with TBST, and then incubated with HRP-conjugated secondary antibodies for 1 h at room temperature, followed by another five washes with TBST. Protein bands were visualized using an enhanced chemiluminescence reagent and imaged on a Tanon automatic chemiluminescence image analysis system (Tanon, Shanghai, China). Rabbit antibodies, including anti-BAX, anti-Bcl-2, anti-Cl-Cas3, anti-phospho-PI3K-P85α, anti-phospho-Akt-S473, anti-mTOR, anti-phospho-mTOR-S2448, anti-PKM2, anti-GLUT1, anti-PFKFB2, anti-HK2, anti-LDHA, anti-PDK1, anti-COX5B, anti-ATP5G1, anti-MFN1, anti-FIS1, and anti-OPA1, were used at a 1:1,000 dilution. Rabbit antibodies, including anti-SDHB and anti-NDUFB9, were used at a 1:2,000 dilution. Rabbit antibodies, including anti-PI3K and anti-Akt, were used at a 1:10,000 dilution. Mouse antibodies, including anti-NDUFB8, anti-UQCRC1, and anti-α-Tubulin, were used at a 1:5,000 dilution. HRP-conjugated goat anti-rabbit IgG and goat anti-mouse IgG, both at a 1:5,000 dilution, were used as secondary antibodies. Finally, the blots were quantified using ImageJ software (GE Healthcare Life Sciences, UK).

### Immunofluorescence assay

PK-15 cells were cultured on coverslips and infected with RH-RFP tachyzoites at an MOI of 5:1 for 0, 12, 24, and 36 h. After the specified infection times, the cell culture medium was removed, and pre-warmed (37°C) Mito-Tracker Green staining solution (200 nM) was added, following the manufacturer’s instructions. The cells were incubated for 30 min, after which the staining solution was discarded, and fresh pre-warmed (37°C) cell culture medium was added. The cells were then observed using a laser confocal microscope (LCM; Leica TCS SP8 STED, Wetzlar, Germany).

### Cell viability assay

Cell viability was detected by using the Cell Counting Kit-8 (CCK-8) reagent. Briefly, PK-15 cells (5,000 cells/well) were seeded in 96-well plates and cultured at 37°C with 5% CO_2_ for 12 h. Afterward, different concentrations of LY294002 (0–100 µM) were applied to the cells for 24 h. Absorbance at 450 nm of each well cell was measured using a microplate absorbance reader. Data were compiled from three independent experiments.

### Effects of LY294002 on intracellular *T. gondii*

Monolayer PK-15 cells were seeded in 24-well plates, and RH-RFP tachyzoites were added at an MOI of 1. The cells were incubated at 37°C in a 5% CO_2_ atmosphere for 3 h. Afterward, the medium containing extracellular parasites was removed, and fresh medium containing LY294002 at concentrations of 0, 2, 5, 8, and 10 µM, along with 0.2% DMSO as a positive control, was added. Following a 24 h incubation at 37°C, the growth of RH-RFP was observed using a fluorescence microscope. The growth rate was analyzed using Image-Pro Express (30).

### Intracellular proliferation of *T. gondii*

A total of 1 × 10^5^ freshly egressed RH-RFP tachyzoites were allowed to infect PK-15 monolayers for 3 h, after which the medium was replaced with fresh medium containing LY294002 at concentrations of 0, 2, 5, 8, and 10 µM, along with 0.2% DMSO. The infected parasites were co-cultured at 37°C in a 5% CO_2_ atmosphere for 24 h. A total of 100 parasitophorous vacuoles (PVs) were randomly selected, and the number of parasites in each vacuole was counted. The results represent data from three independent experiments (31).

### Statistical analysis

In this study, statistical data were representative of ≥3 independent experiments and presented as the mean ± standard deviation (SD). Comparisons between two groups were analyzed using the *t*-test, while comparisons among multiple groups were analyzed using one-way analysis of variance. Differences were considered statistically significant at a *P* value of <0.05. All statistical analyses were carried out using GraphPad Prism 8.0 software (GraphPad Software, San Diego, CA, USA).

## RESULTS

### *T. gondii* infection induces mitochondrial damage and reduces ΔΨm

**Fig 1:**
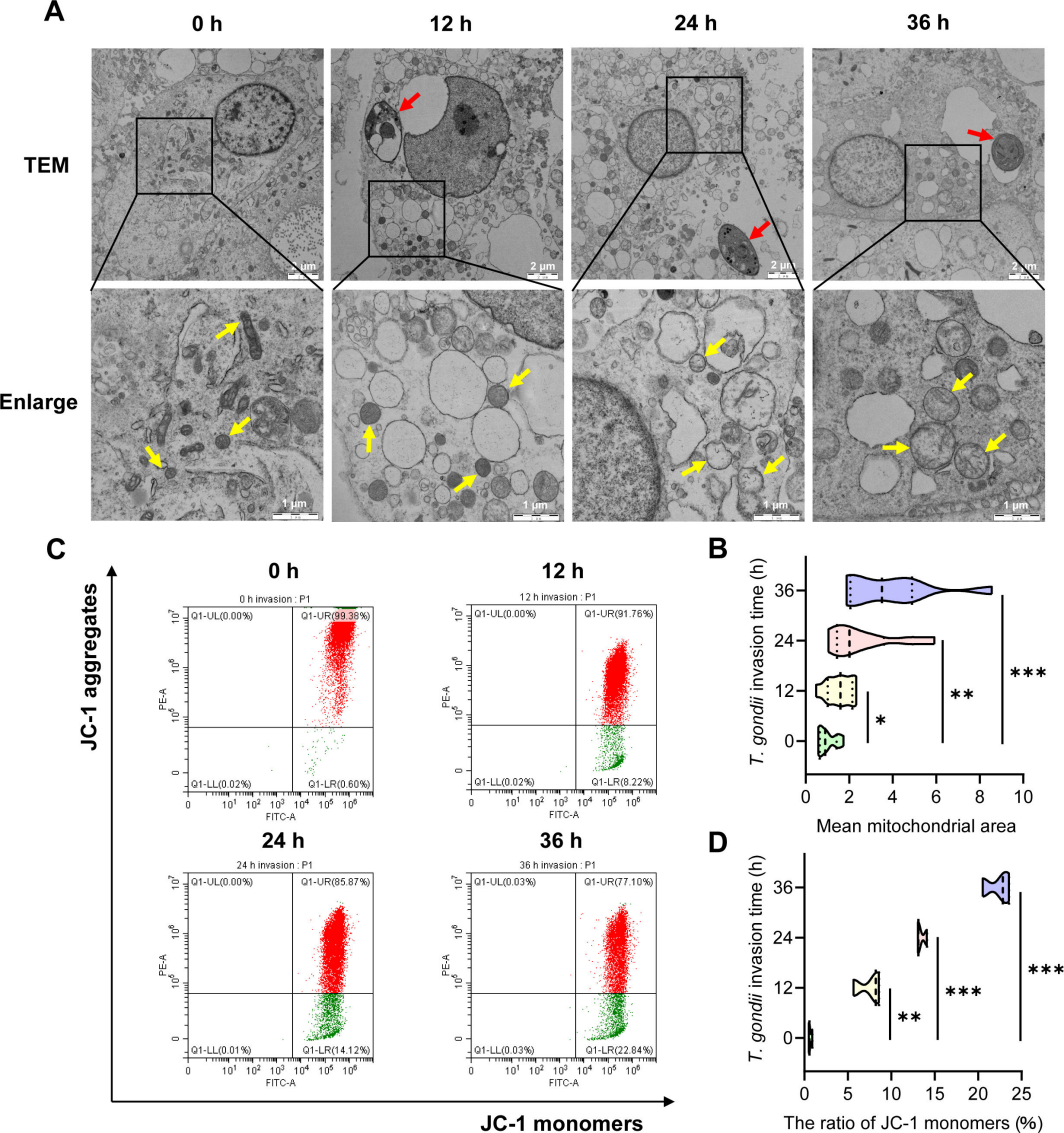
*T. gondii* (MOI = 5) induces mitochondrial damage and reduces mitochondrial membrane potential (ΔΨm) in PK-15 cells. (**A**) Morphological changes in mitochondria of PK-15 cells at different time points (0, 12, 24, and 36 h) post-infection. Scale bar, 2 μm. Red arrows indicate *T. gondii* tachyzoites within the cells, and yellow arrows indicate mitochondria of PK-15 cells before and after infection. Insets show higher magnification of the outlined areas, with a scale bar of 1 μm. (**B**) Average mitochondrial area of PK-15 cells at different time points post-*T. gondii* infection. Data were normalized to the 0 h group. (**C**) ΔΨm in PK-15 cells at different time points post-infection. (**D**) Ratio of JC-1 monomers in PK-15 cells at various time points after *T. gondii* infection. All data are presented as the mean ± SD from at least three independent biological replicates. *P < 0.05, **P < 0.01, and ***P < 0.001.

TEM results revealed that mitochondria in uninfected cells exhibited an oval or rod-shaped morphology with a double-membrane structure and inward-folded cristae, while *T. gondii*-infected cells displayed swollen, fragmented, and oval-shaped mitochondria with disappeared cristae (Fig. 1A). Furthermore, the average mitochondrial area gradually increased with prolonged *T. gondii* infection (Fig. 1B). JC-1, a widely used fluorescent probe for detecting ΔΨm, forms aggregates in the mitochondrial matrix and emits red fluorescence when ΔΨm is high, whereas it remains in its monomeric form and emits green fluorescence when ΔΨm is low. A decrease in ΔΨm is also an early hallmark of apoptosis. Flow cytometry (FCM) results demonstrated that, with extended *T. gondii* infection, ΔΨm in PK-15 cells gradually decreased, as indicated by an increasing shift from red to green fluorescence in JC-1 staining (Fig. 1C and D), further suggesting that *T. gondii* infection induces mitochondrial damage in PK-15 cells in a time-dependent manner.

### *T. gondii* infection induces mitochondrial fusion and apoptosis in PK-15 cells

**Fig 2:**
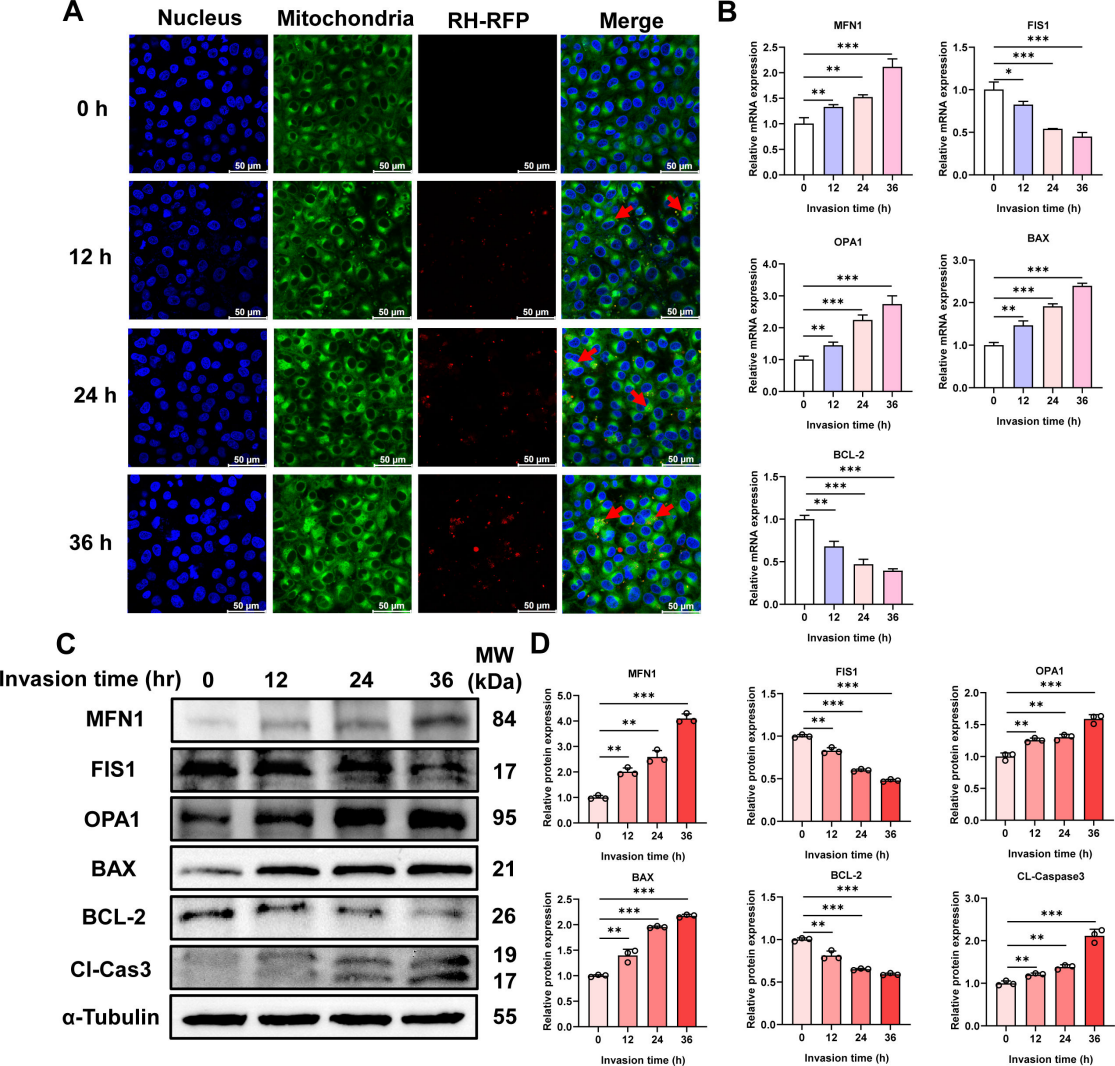
*T. gondii* (MOI = 5) infection induces mitochondrial fusion and initiates apoptosis in PK-15 cells. (**A**) Mitochondrial aggregation in PK-15 cells at different time points (0, 12, 24, and 36 h) post-*T. gondii* infection. Scale bar, 50 μm. Red arrows indicate *T. gondii* tachyzoites inside the cells. (**B**) mRNA levels of MFN1, FIS1, OPA1, BAX, and BCL-2, and protein (**C**) levels of MFN1, FIS1, OPA1, BAX, BCL-2, and cleaved caspase-3 (Cl-Cas3) at different time points post-infection in PK-15 cells. (**D**) Quantitative analysis of gray value of panel **C**. All data are presented as the mean ± SD from at least three independent biological replicates. *P < 0.05, **P < 0.01, and ***P < 0.001.

To further investigate the effect of *T. gondii* infection on mitochondrial morphology in cells, immunofluorescence assay (IFA) was conducted to examine mitochondrial alterations in PK-15 cells at different time points post-infection. The results revealed that as the infection time progressed, mitochondria labeled with Mito-Tracker exhibited aggregation, and the fluorescence intensity increased, indicating that *T. gondii* infection induces mitochondrial fusion in the cells (Fig. 2A). qPCR (Fig. 2B) and western blotting (WB; Fig. 2C and D) analysis provide supportive and complementary evidence, demonstrating that the expression levels of mitochondrial fusion factors MFN1 and OPA1 gradually increased, while the expression of the mitochondrial fission factor FIS1 gradually decreased with prolonged infection time. Additionally, consistent with *T. gondii*-induced mitochondrial damage, the decrease in ΔΨm, and previous research findings (2), *T. gondii* infection led to an upregulation of the pro-apoptotic factor BAX at both the mRNA (Fig. 2B) and protein levels (Fig. 2C and D), accompanied by a concomitant downregulation of the anti-apoptotic factor BCL-2 at the mRNA (Fig. 2B) and protein levels (Fig. 2C and D), as well as an enhanced expression of the apoptosis executioner protein Cl-Cas3 at the protein level (Fig. 2C and D). These results collectively suggest that *T. gondii* infection triggers mitochondrial-dependent apoptosis in PK-15 cells.

### *T. gondii* infection reduces NAD^+^/NADH ratio, ATP production, and OXPHOS in PK-15 cells

**Fig 3:**
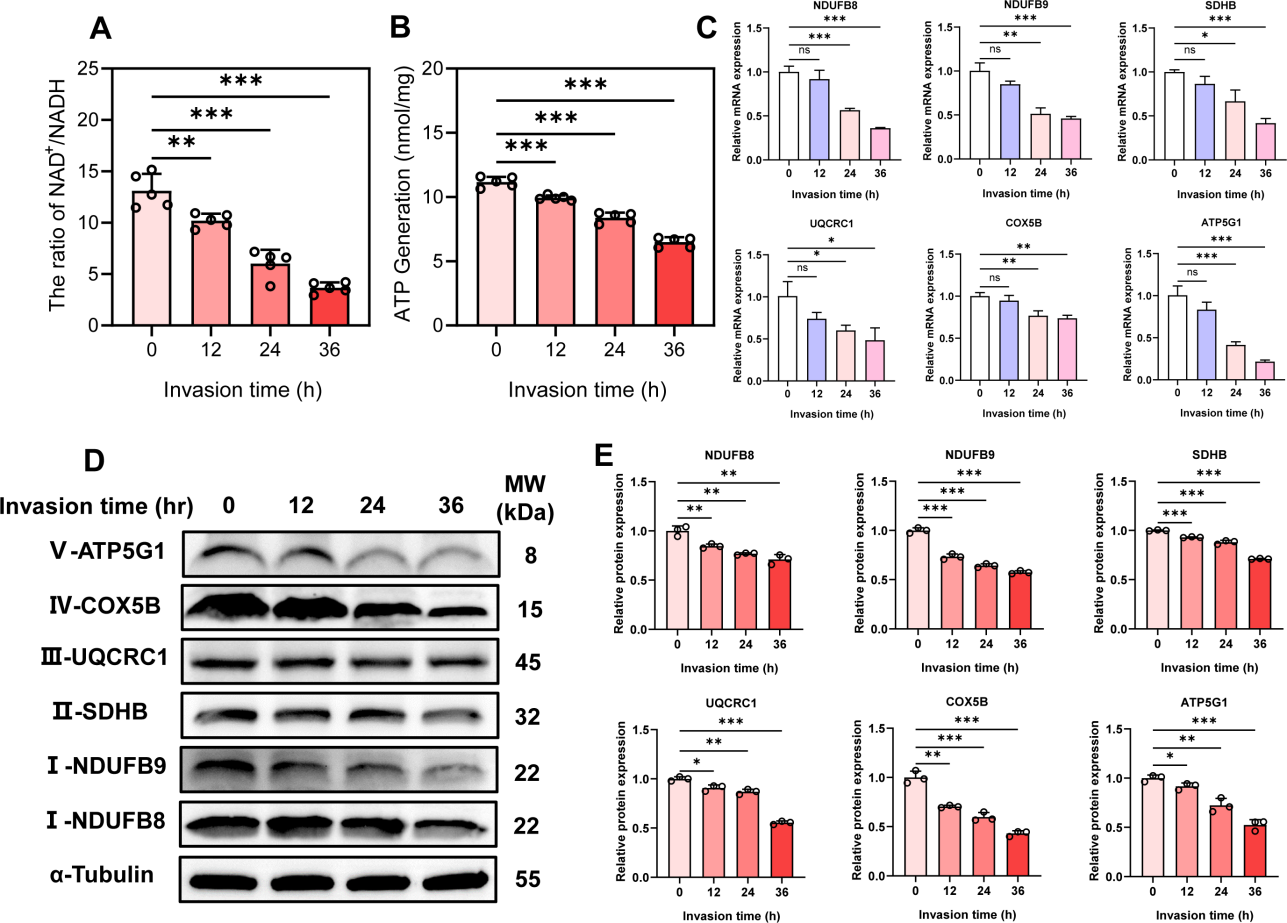
*T. gondii* (MOI = 5) infection reduces NAD^+^/NADH ratio, ATP production, and inhibits the expression of OXPHOS-related molecules in PK-15 cells. (**A**) NAD^+^/NADH ratio and (**B**) ATP production in PK-15 cells at different time points (0, 12, 24, and 36 h) post-*T. gondii* infection. (**C**) *T. gondii* infection induces a time-dependent downregulation of NDUFB8, NDUFB9, SDHB, UQCRC1, COX5B, and ATP5G1 mRNAs and (**D**) proteins in PK-15 cells. (**E**) Quantitative analysis of gray value of panel **D**. All data are presented as the mean ± SD from at least three independent biological replicates. *P < 0.05, **P < 0.01, and ***P < 0.001; ns, not significant.

The results of the kit assay demonstrated that *T. gondii* infection reduced the NAD^+^/NADH ratio (Fig. 3A) and ATP production (Fig. 3B) in a time-dependent manner. Specifically, ATP levels decreased by approximately 42% at 36 h post-infection compared with uninfected controls. Mitochondrial dysfunction, along with the downregulation of OXPHOS-related genes, emerged as a key characteristic during *T. gondii* infection. To further investigate this, qPCR and WB analysis were performed to assess the expression levels of mitochondrial OXPHOS enzyme complexes at different time points post-infection in PK-15 cells, including Complex I (NDUFB8 and NDUFB9), Complex II (SDHB), Complex III (UQCRC1), Complex IV (COX5B), and Complex V (ATP5G1). The results showed that *T. gondii* infection led to a time-dependent downregulation of OXPHOS-related genes (Fig. 3C) and proteins in PK-15 cells (Fig. 3D and E). These findings suggest that *T. gondii* infection causes mitochondrial damage and impairs the OXPHOS process in PK-15 cells.

### *T. gondii* infection promotes energy metabolism and glycolysis in PK-15 cells

**Fig 4:**
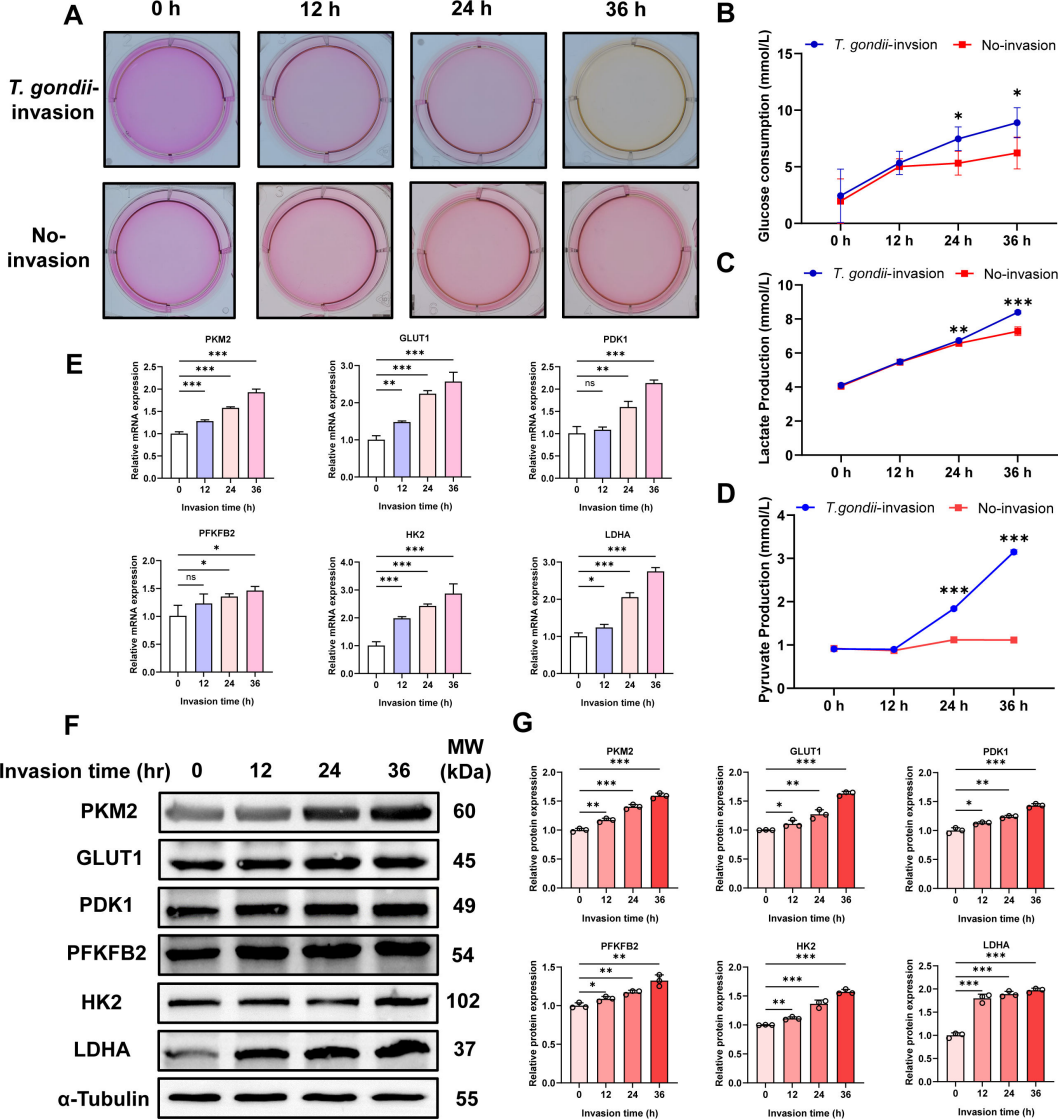
*T. gondii* (MOI = 5) infection promotes energy metabolism and glycolysis in PK-15 cells. (**A**) Media acidification of PK-15 cells with or without *T. gondii* infection at different time points (0, 12, 24, and 36 h). (**B**) Glucose consumption, (**C**) lactate production, and (**D**) pyruvate production in PK-15 cells at different time points before and after infection. (**E**) Relative expression levels of PKM2, GLUT1, PDK1, PFKFB2, HK2, and LDHA mRNAs, and (**F**) protein levels in PK-15 cells at different time points post-infection. (**G**) Quantitative analysis of gray value of panel **F**. All data are presented as the mean ± SD from at least three independent biological replicates. *P < 0.05, **P < 0.01, and ***P < 0.001; ns, not significant.

In the experiment, we observed an intriguing phenomenon: after inoculating *T. gondii* tachyzoites onto PK-15 cells at an MOI of 5:1, the culture medium in the six-well plate turned yellow faster than in uninfected cells as the infection time progressed (Fig. 4A). Phenol red in the culture medium acts as a pH indicator, turning yellow due to the accumulation of acidic substances, indirectly reflecting cellular metabolic activity (32). Additionally, measurements of glucose consumption and lactate and pyruvate production in PK-15 cells at various time points before and after infection indicated that, as the infection progressed, glucose uptake (Fig. 4B) and lactate (Fig. 4C) and pyruvate production (Fig. 4D) were elevated in *T. gondii*-infected cells compared with uninfected controls. These experimental results strongly suggest that *T. gondii* infection enhances the energy metabolism of PK-15 cells in a time-dependent manner. Further qPCR analysis demonstrated that the expression of glycolysis-related genes, including *PKM2*, *GLUT1*, *PDK1*, *PFKFB2*, *HK2*, and *LDHA*, was progressively upregulated in PK-15 cells following *T. gondii* infection in a time-dependent manner (Fig. 4E). Consistently, WB results confirmed increased protein levels of these glycolysis-associated molecules (Fig. 4F and G). In summary, the analysis of metabolite levels and the results from qPCR and WB experiments collectively indicate that *T. gondii* infection promotes glycolytic metabolism in PK-15 cells.

### *T. gondii* infection activates the PI3K/Akt/mTOR pathway in PK-15 cells

**Fig 5:**
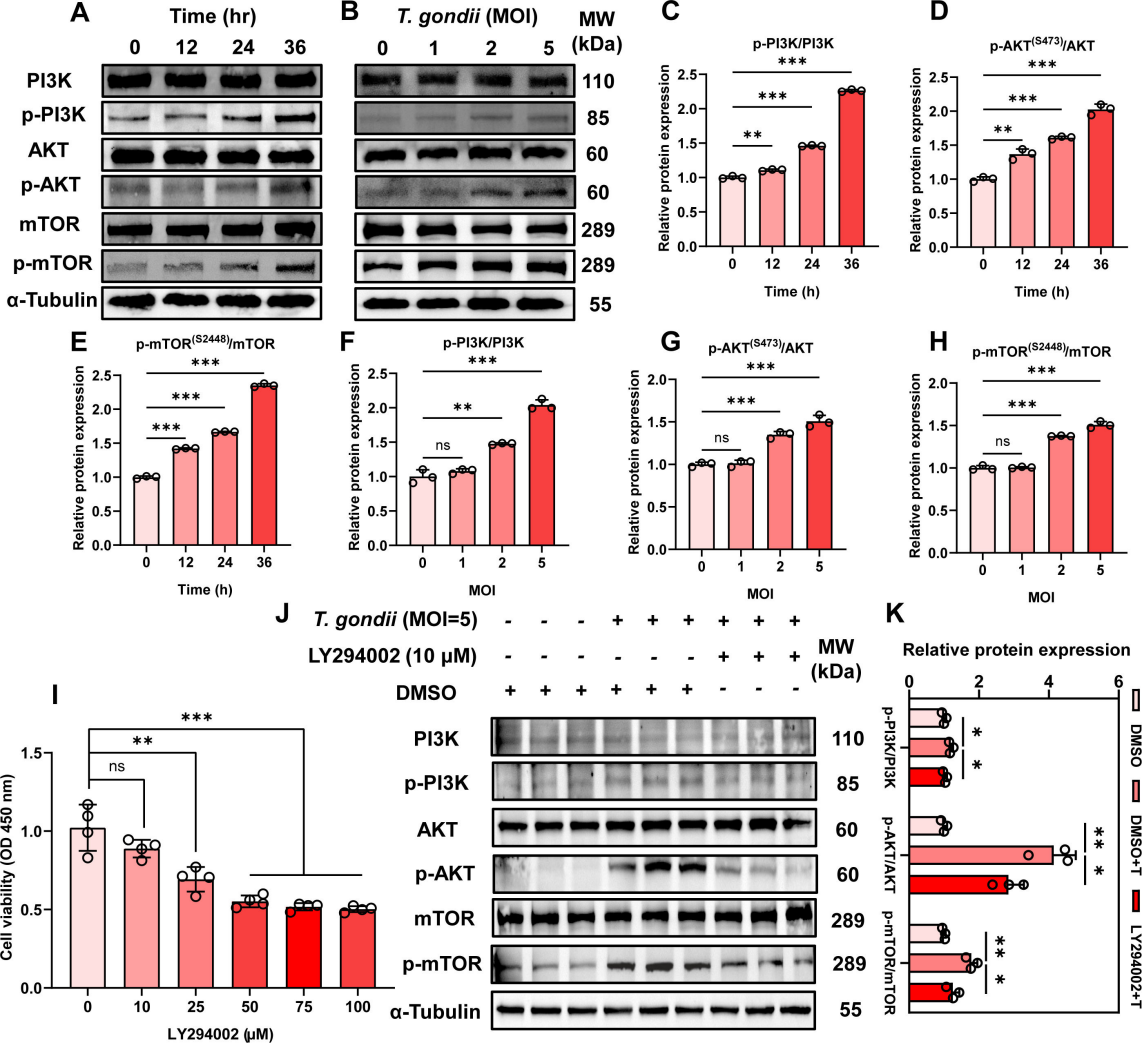
*T. gondii* infection activates the PI3K/Akt/mTOR signaling pathway in PK-15 cells. (**A**) Activation of the PI3K/Akt/mTOR pathway was observed at different time points (0, 12, 24, and 36 h) post-infection at an MOI of 5, and (**B**) at different MOIs (0, 1, 2, and 5) after 24 h. (**C–E**) Quantitative analysis of gray value of panel **A**. (**F–H**) Quantitative analysis of gray value of panel **B**. (**I**) CCK-8 assay was used to determine the working concentration of the PI3K/Akt/mTOR pathway-specific inhibitor LY294002 in PK-15 cells. (**J**) LY294002 (10 μM) inhibits the activation of the PI3K/Akt/mTOR pathway induced by *T. gondii* infection (MOI = 5) for 24 h in PK-15 cells. (**K**) The quantitative analysis of gray value of panel **J**. All data are presented as the mean ± SD from at least three independent biological replicates. *P < 0.05, **P < 0.01, and ***P < 0.001; ns, not significant.

To clarify the regulatory role of the PI3K/Akt/mTOR pathway in *T. gondii*-induced metabolic reprogramming, we investigated the activation of this pathway in PK-15 cells infected with *T. gondii* at different time points (0, 12, 24, and 36 h; Fig. 5A) and at various doses (MOI = 0, 1, 2, 5; Fig. 5B) for 24 h using WB analysis. The experimental results indicated that *T. gondii* infection activates the PI3K/Akt/mTOR signaling pathway in PK-15 cells in a time-dependent (Fig. 5C through E) and dose-dependent (Fig. 5F through H) manner. To further confirm this, we used LY294002, a specific inhibitor of the PI3K/Akt/mTOR pathway. First, a CCK-8 assay was performed to establish the working concentration of LY294002 in PK-15 cells, determining that a maximum concentration of 10 μM for 24 h did not affect PK-15 cell proliferation (Fig. 5I). Subsequently, uninfected PK-15 cells, cells infected with *T. gondii* (MOI = 5), and cells pretreated with 10 μM LY294002 prior to *T. gondii* infection (MOI = 5) were collected 24 h post-infection. WB analysis demonstrated that *T. gondii* infection activated the PI3K/Akt/mTOR signaling pathway in PK-15 cells, whereas LY294002 effectively suppressed *T. gondii*-induced activation of this pathway (Fig. 5J and K).

### LY294002 inhibits *T. gondii*-induced metabolic reprogramming in PK-15 cells

**Fig 6:**
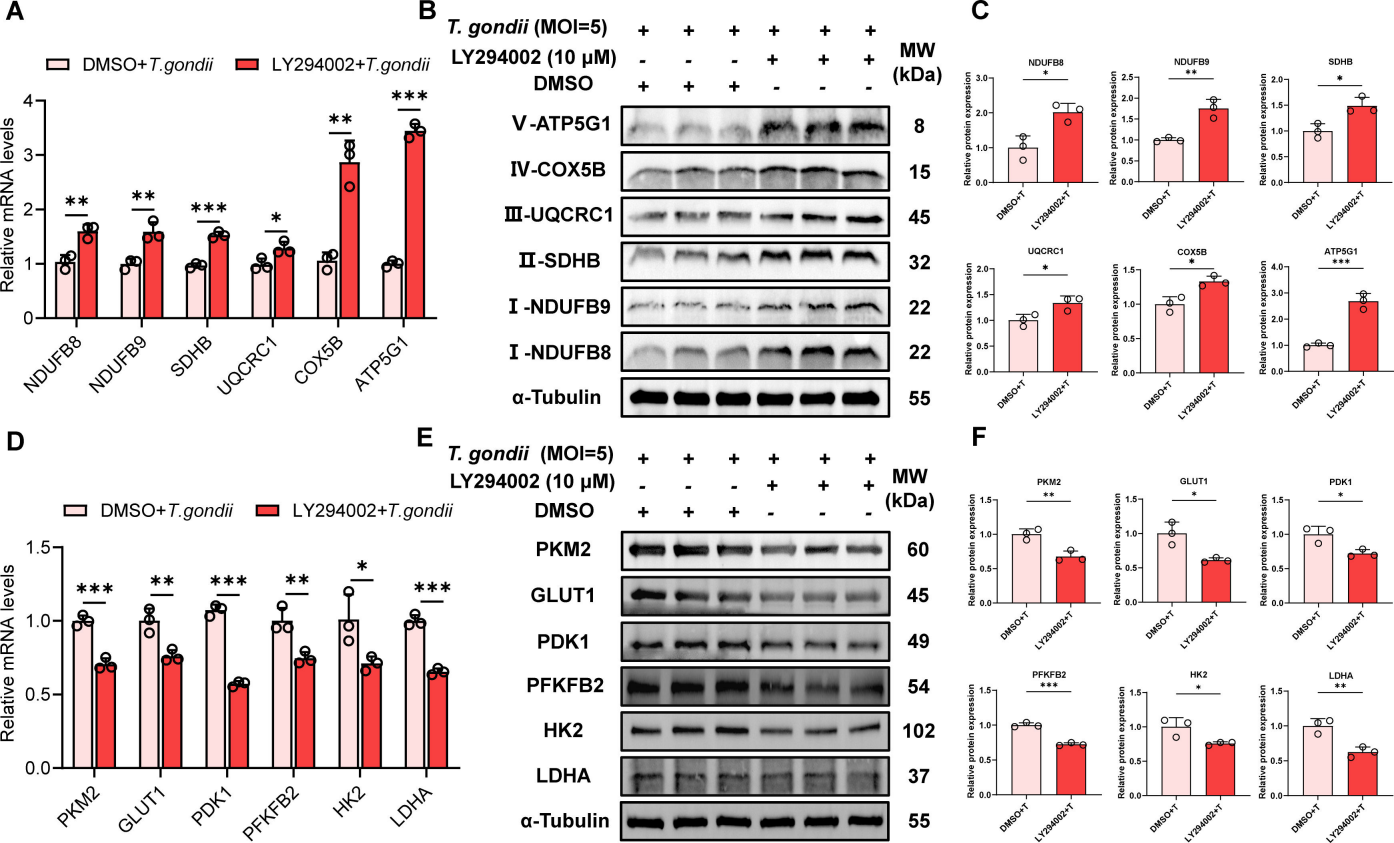
LY294002 (10 μM) reverses *T. gondii* (MOI = 5)-induced glycolysis increase and OXPHOS decrease in PK-15 cells. (**A**) mRNA and (**B**) protein expression levels of OXPHOS-related molecules (NDUFB8, NDUFB9, SDHB, UQCRC1, COX5B, and ATP5G1) were measured 24 h after *T. gondii* infection, with or without LY294002 treatment. (**C**) Quantitative analysis of gray value of panel **B**. (**D**) mRNA and (**E**) protein expression levels of glycolysis-related molecules (PKM2, GLUT1, PDK1, PFKFB2, HK2, and LDHA) were measured 24 h after *T. gondii* infection, with or without LY294002 treatment. (**F**) Quantitative analysis of gray value of panel **E**. All data are presented as the mean ± SD from at least three independent biological replicates. *P < 0.05, **P < 0.01, and ***P < 0.001.

To further investigate the role of the PI3K/Akt/mTOR pathway in *T. gondii* infection-induced metabolic reprogramming in PK-15 cells, qPCR and WB were used to assess the effects of LY294002 on the expression of glycolysis- and OXPHOS-related molecules in infected cells. The results indicated that LY294002 inhibited the *T. gondii*-induced increase in glycolysis-related gene (Fig. 6A) and protein (Fig. 6B and C) expression and suppressed the decrease in OXPHOS-related gene (Fig. 6D) and protein (Fig. 6E and F) expression. These experimental findings suggest that the PI3K/Akt/mTOR pathway-specific inhibitor LY294002 can reverse *T. gondii*-induced metabolic reprogramming in PK-15 cells.

### LY294002 regulates *T. gondii*-induced mitochondrial function changes in PK-15 cells

**Fig 7:**
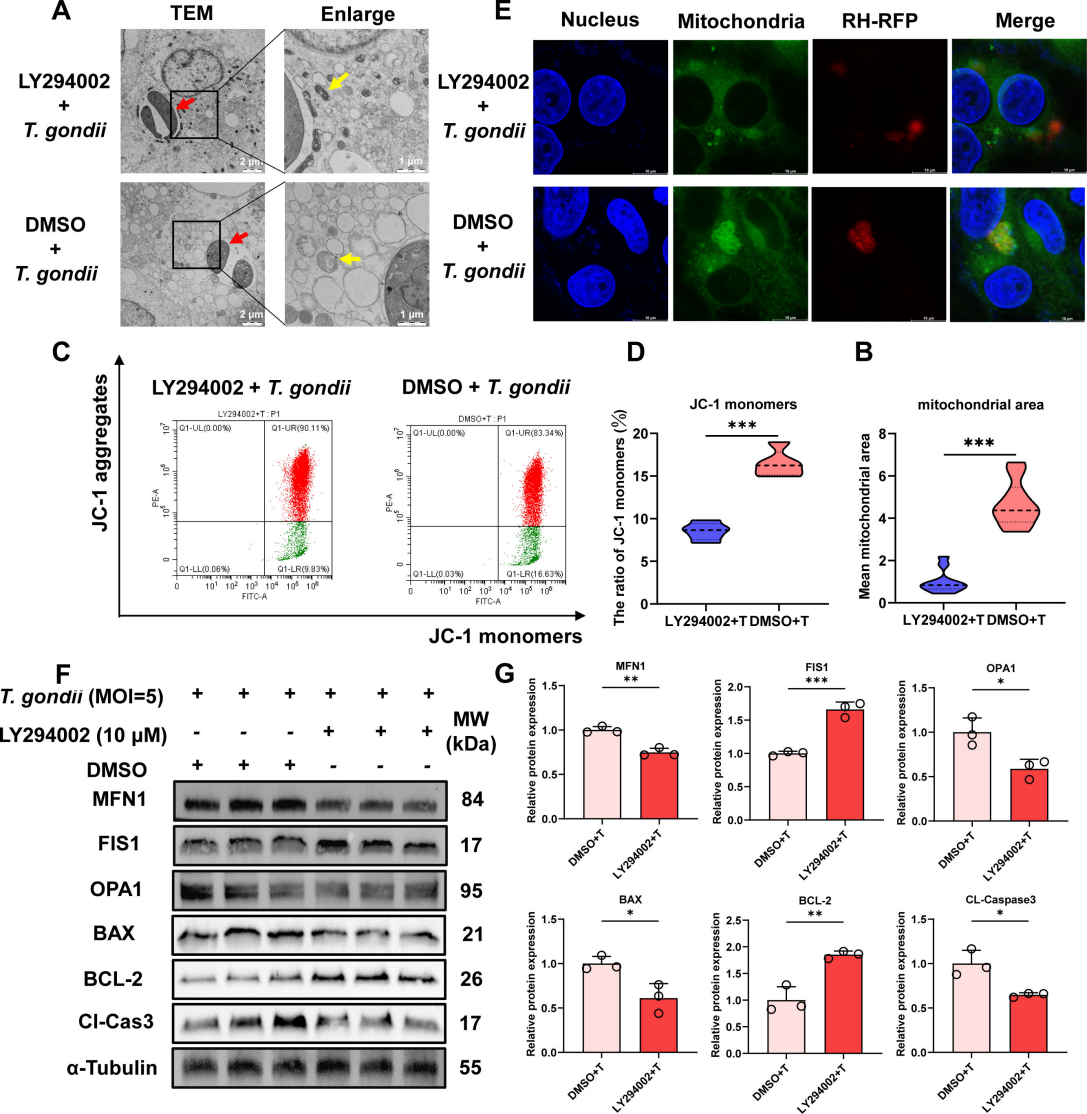
LY294002 (10 μM) inhibits *T. gondii* (MOI = 5)-induced mitochondrial damage and fusion. (**A**) LY294002 provides protective effects against mitochondrial damage induced by *T. gondii* infection. Scale bar, 2 μm. Red arrows indicate *T. gondii* tachyzoites inside the cells, while yellow arrows indicate mitochondria of PK-15 cells. Insets providing a higher magnification (scale bar, 1 μm). (**B**) Average mitochondrial area of PK-15 cells at 24 h post-*T. gondii* infection, with or without LY294002 treatment. (**C**) Mitochondrial membrane potential in PK-15 cells at 24 h post-infection, with or without LY294002 treatment. (**D**) Ratio of JC-1 monomers in PK-15 cells. (**E**) LY294002 inhibits *T. gondii*-induced mitochondrial fusion in PK-15 cells. Scale bar, 50 μm. (**F**) LY294002 reverses *T. gondii*-induced changes in mitochondrial fusion and apoptosis-related proteins in PK-15 cells after 24 h of infection. (**G**) Quantitative analysis of gray value of panel **F**. All data are presented as the mean ± SD from at least three independent biological replicates. *P < 0.05, **P < 0.01, and ***P < 0.001.

To determine whether *T. gondii*-induced mitochondrial function changes in PK-15 cells occur through the PI3K/Akt/mTOR pathway, we treated the cells with 10 μM LY294002 and then infected them with *T. gondii* at an MOI of 5:1. TEM results demonstrated that LY294002 inhibited *T. gondii* infection-induced mitochondrial swelling, cristae disappearance, and reduced the average mitochondrial area (Fig. 7A and B). FCM results revealed that LY294002 attenuated the *T. gondii* infection-induced decrease in ΔΨm, as evidenced by the inhibition of JC-1 monomer increase (Fig. 7C and D). These findings suggest that LY294002 exerts a protective effect against *T. gondii* infection-induced mitochondrial damage. Additionally, IFA results indicated that LY294002 inhibited *T. gondii* infection-induced mitochondrial fusion (Fig. 7E). Furthermore, the WB results provided additional evidence that LY294002 suppressed *T. gondii* infection-induced mitochondrial fusion and mitochondrial damage (Fig. 7F and G).

### LY294002 inhibits energy metabolism in *T. gondii*-infected PK-15 cells

**Fig 8:**
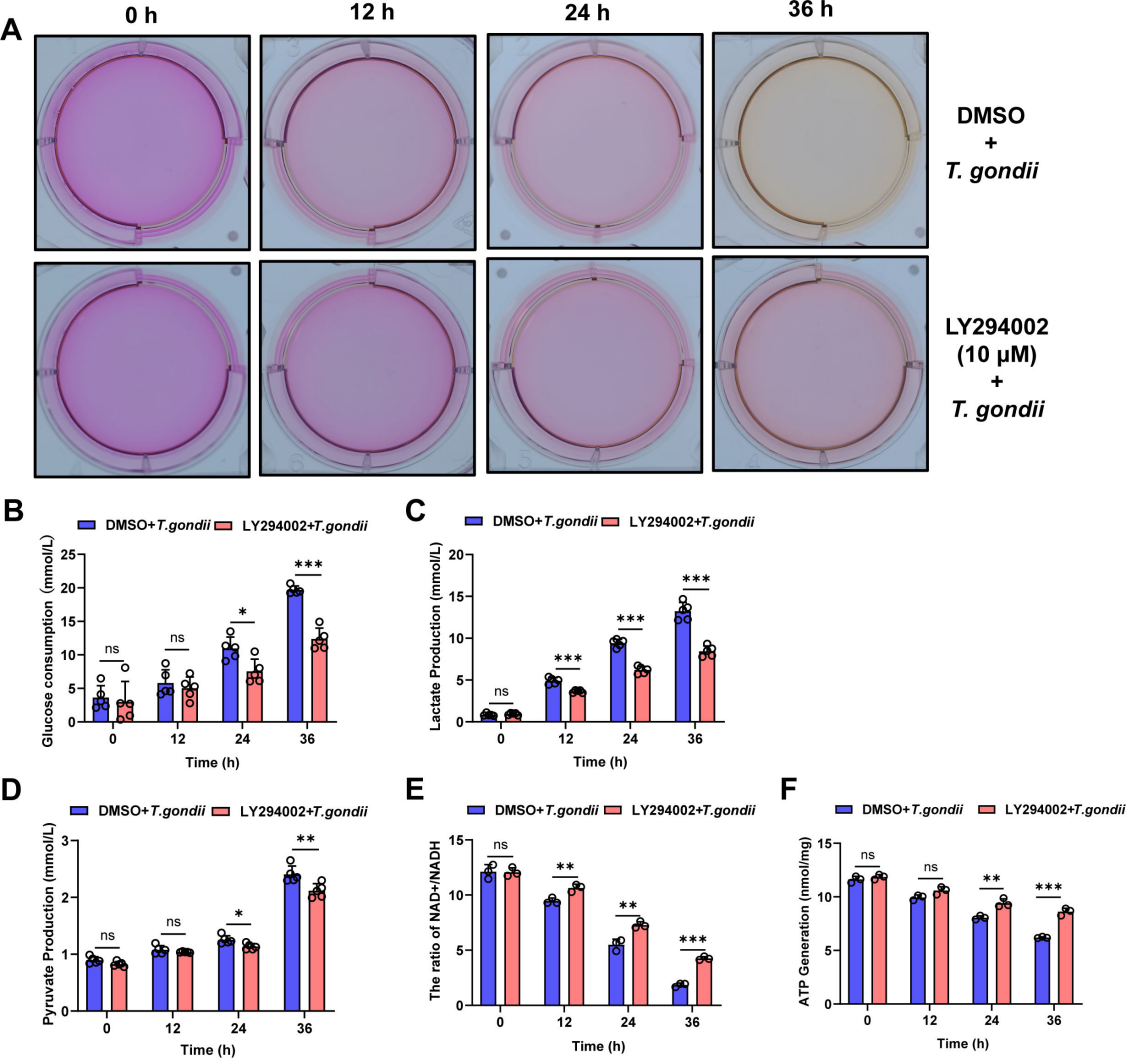
LY294002 (10 μM) inhibits *T. gondii* (MOI = 5)-induced host cell metabolic phenotype changes. (**A**) Comparison of culture medium color at different time points (0, 12, 24, and 36 h) after *T. gondii* infection in PK-15 cells, with and without LY294002 treatment. (**B**) Effect of LY294002 on glucose consumption, (**C**) lactate production, (**D**) pyruvate production, (**E**) NAD^+^/NADH ratio, and (**F**) ATP production at different time points post-infection in PK-15 cells. All graph data are expressed as the mean ± SD of at least three biological replicates per group. *P < 0.05, **P < 0.01, and ***P < 0.001; ns, not significant.

Subsequently, we investigated the effects of LY294002 on the metabolic reprogramming phenotype induced by *T. gondii* infection in PK-15 cells. The results showed that LY294002 inhibited the yellowing of the culture medium caused by *T. gondii* infection (Fig. 8A). Corresponding commercial kits were used to measure glucose consumption, lactate and pyruvate production, NAD^+^/NADH ratio, and ATP generation. The findings indicated that LY294002 suppressed the increase in glucose uptake (Fig. 8B) induced by *T. gondii* infection, inhibited the production of lactate (Fig. 8C) and pyruvate (Fig. 8D), and elevated the NAD^+^/NADH (Fig. 8E) ratio and ATP levels (Fig. 8F). This further suggests that *T. gondii*-induced metabolic reprogramming in PK-15 cells is mediated through the activation of the PI3K/Akt/mTOR pathway.

### LY294002 inhibits the replication and proliferation of *T. gondii* in PK-15 cells

**Fig 9:**
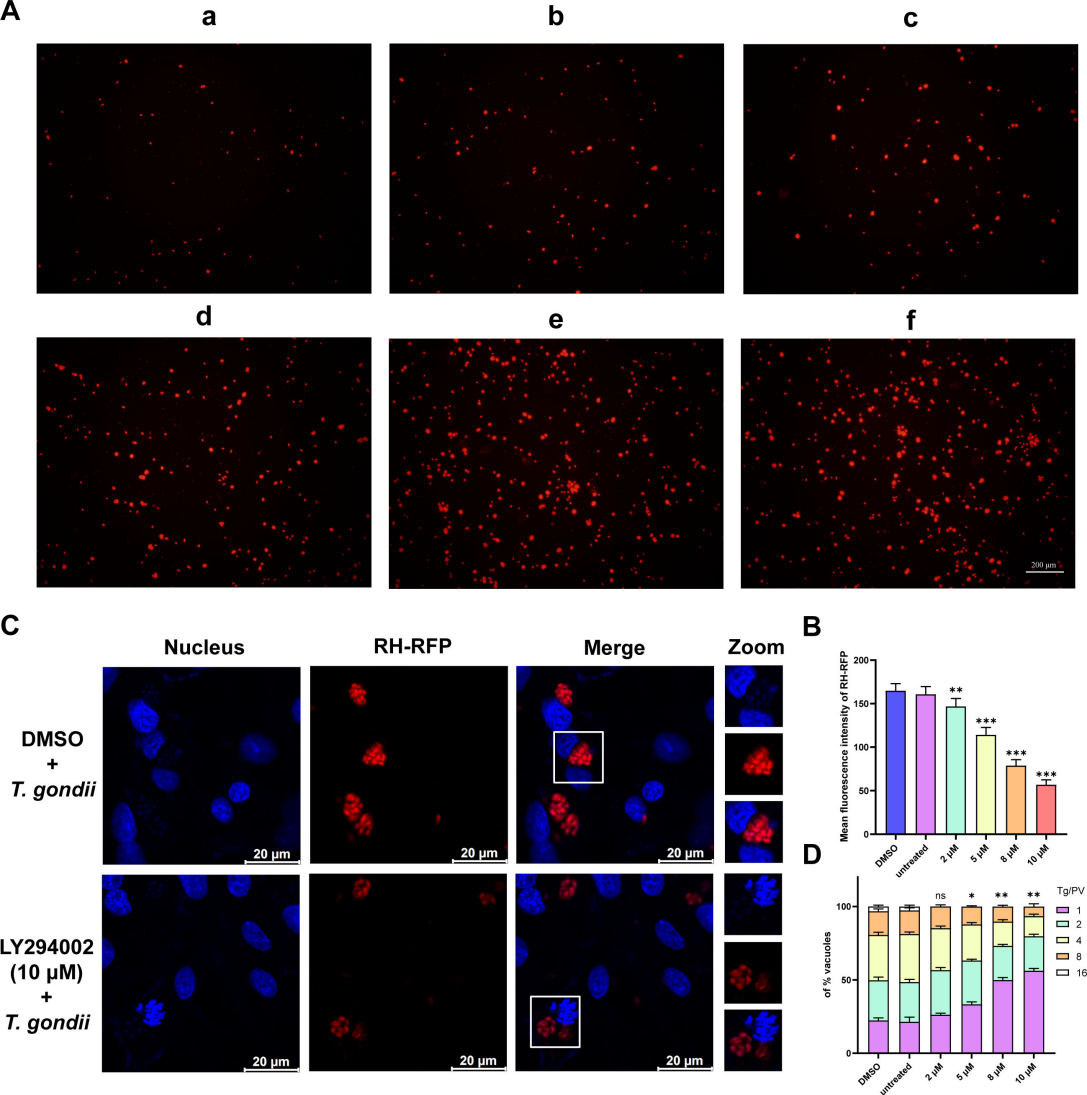
LY294002 inhibits the replication and proliferation of *T. gondii* within host cells. (**A**) Anti-*T*. *gondii* activity of LY294002 was evaluated by RH-RFP growth assay. The growth rate was calculated by measuring fluorescence intensity. (a–e) PK-15 cells were infected with RH-RFP and treated with different concentrations of LY294002 for 24 h: (a) 10 µM, (b) 8 µM, (c) 5 µM, (d) 2 µM, (e) untreated, and (f) DMSO as a positive control. (**B**) Statistical analysis of the growth rates in different treatment groups. All images were observed under 200× magnification; scale bar = 200 μm. (**C**) Effect of LY294002 (10 μM) on the *T. gondii* intracellular proliferation for 24 h. (**D**) Intracellular proliferation was assessed after *T. gondii* tachyzoites were treated with different concentrations of LY294002 for 24 h. Numbers 1, 2, 4, 8, and 16 represent the number of tachyzoites in each PV. Data are presented as the mean ± SD from three independent experiments, with three technical replicates per experiment. *P < 0.05, **P < 0.01, and ***P < 0.001; ns, not significant.

The results from the parasite replication experiment demonstrated that, with increasing concentrations of LY294002, the replication of intracellular RH-RFP tachyzoites gradually decreased. This was evidenced by the reduction in the mean fluorescence intensity of RH-RFP tachyzoites within the same field of view as the concentration of LY294002 increased (Fig. 9A and B). Additionally, the inhibitory effect of LY294002 on *T. gondii* intracellular proliferation was evaluated through replication assays on PK-15 cells. One hundred PVs were randomly selected to count the number of tachyzoites. As shown in Fig. 9C and D, nearly 80% of PVs contained one or two tachyzoites, with a maximum of eight tachyzoites per PV in the 10 μM LY294002 group, indicating that the proliferation rate was significantly inhibited by LY294002. Compared to the DMSO and untreated groups, the LY294002-treated groups showed a significantly reduced proliferation rate in a dose-dependent manner over 24 h.

## DISCUSSION

*T. gondii* is an obligate intracellular parasite whose intracellular growth relies on carbon sources such as glucose and glutamine provided by the host. Studies have shown that glucose concentration directly influences the parasite’s growth rate. *T. gondii* proliferates rapidly under high-glucose conditions but exhibits slow growth under glucose-deficient conditions, as glucose metabolites enter the tricarboxylic acid (TCA) cycle to promote energy production and biosynthesis (33). Under normal conditions, host cells maintain energy supply through mitochondrial OXPHOS. However, *T. gondii*-infected cells sustain glycolytic metabolism even under aerobic conditions (14, 15). This demonstrates the ability of *T. gondii* to reprogram metabolic pathways of the host cells. Nevertheless, the molecular mechanisms underlying this parasite-mediated metabolic reprogramming remain unclear.

Our previous studies have discovered that *T. gondii* promotes host cell apoptosis through the mitochondrial pathway (2, 34), suggesting that the pathogenic effects caused by *T. gondii* proliferation may be closely associated with mitochondrial dysfunction (35). Following the invasion, *T. gondii* forms a PV within the host cell to support its growth and replication. This process triggers mitochondrial rearrangement and morphological alterations in the host cells, a phenomenon termed host mitochondrial association (HMA) (36–38). The formation mechanism and biological consequences of HMA currently remain unresolved. In this study, electron microscopy and fluorescent probe imaging revealed that *T. gondii* not only disrupted mitochondrial morphology but also altered their spatial distribution, showing that mitochondria clustered around the parasite, which was consistent with previous studies (36, 39–41). Concurrently, this study further revealed that *T. gondii* induced alteration in host mitochondrial dynamics, characterized by promoting mitochondrial fusion and inhibiting fission. Studies have suggested that moderate fusion enhancement serves as a reparative mechanism in host cells under pathological conditions, facilitating mitochondrial network formation and material exchange between damaged mitochondria to adapt to intracellular environmental changes or increased energy demands (42–44). However, excessive fusion disrupts mitochondrial dynamic equilibrium, compromising mitochondrial integrity and function. This study demonstrated, through multiple perspectives including mitochondrial morphology, NAD^+^/NADH ratio, ATP production, and ΔΨm, that *T. gondii* infection severely disrupted host mitochondrial homeostasis and energy output, which were critical for maintaining cellular energy balance. ATP depletion redirects cell fate from apoptosis to necrosis and perturbs caspase-dependent pathways (45); consequently, even modest reductions in ATP are biologically meaningful, impairing mitochondrial function and compromising cellular homeostasis (46). During this process, mitochondrial OXPHOS was significantly suppressed, confirming the parasite’s ability to impair host mitochondrial function. OXPHOS is mediated by five multi-subunit complexes embedded within the inner mitochondrial membrane, which sequentially couple electron transfer with proton translocation to drive ATP synthesis (47, 48). Electrons derived from NADH or succinate are conveyed through complexes I–IV via ubiquinone and cytochrome c to molecular oxygen, with complexes I, III, and IV concomitantly generating the proton gradient required for energy conversion (49–52). Complex V (ATP synthase) subsequently exploits this proton-motive force to drive phosphorylation of ADP to ATP (53). Collectively, these processes are central to sustaining mitochondrial bioenergetics (54), and their selective suppression by *T. gondii* reveals a potential mechanism by which the parasite reprograms host energy metabolism to its advantage. Although prior studies have highlighted that OXPHOS is essential for maintaining the ATP level in the fast-growing tachyzoite stage (55), and have been considered to be promising anti-parasitic drug targets through interfering with this pathway (56–58), how the parasite manipulates host energy metabolism and mitochondrial OXPHOS remains poorly understood. Only limited transcriptomic studies have suggested that *T. gondii* downregulates OXPHOS of host cells (8, 35), yet conclusive evidence of parasite-mediated OXPHOS impairment is lacking. Notably and distinctively, our study is the first to identify specific targets of respiratory chain complexes suppressed by *T. gondii*: Complex I (NDUFB8 and NDUFB9), Complex II (SDHB), Complex III (UQCRC1), Complex IV (COX5B), and Complex V (ATP5G1). These targets are critical for electron transport within the respiratory chain and likely represent key pathogenic pathways underlying *T. gondii*-induced mitochondrial dysfunction, as well as pivotal molecular nodes for parasite control over aerobic respiration and redox processes.

Mitochondrial OXPHOS and cytoplasmic glycolysis are the two primary pathways for energy production through glucose metabolism (59). When mitochondrial respiratory chain function is impaired, cells switch to glycolysis for energy production to maintain normal cellular functions. Since OXPHOS is more energy efficient than glycolysis, even minor impairments to the respiratory chain may necessitate a substantial increase in glycolytic activity to compensate for energy deficits. *T. gondii*-induced mitochondrial dysfunction leads to metabolic imbalance and potentially energy insufficiency. Mitochondrial dysfunction and metabolic reprogramming are bidirectional. On the one hand, mitochondrial damage can initiate a compensatory shift toward glycolysis, particularly under conditions of stress or infection (60). On the other hand, enhanced glycolysis can lead to the accumulation of metabolic intermediates and reactive oxygen species, which subsequently exacerbate mitochondrial damage (61). Nevertheless, it remains unclear whether these modified mitochondrial functions occur as a consequence of the host response to *T. gondii* or direct manipulation by the parasite (35). It has been established that during *T. gondii* infection, host cells activate the “Warburg effect,” enhancing glycolytic activity to serve as the primary energy source for rapid intracellular proliferation of the parasites (62–64). In this process, glucose is converted into lactate through a series of enzymatic reactions, releasing ATP. This not only compensates for the energy deficit caused by OXPHOS suppression but also amplifies the biosynthetic activity of *T. gondii* (16), creating an environment that promotes the sustained development of tachyzoites instead of transition to the bradyzoite stage (17). Simultaneously, increased lactate modulates the host immune response within the microenvironment, enabling *T. gondii* to survive and replicate more effectively (65). Furthermore, glycolytic intermediates also contribute to fatty acid synthesis, which is critical for the parasite’s membrane structure and function (66). Therefore, from the perspective of host-parasite interactions, *T. gondii* can adapt to diverse host environments, thus enhancing infection efficiency by inducing the Warburg effect. This metabolic flexibility is a key event and mechanism that allows *T. gondii* to thrive and proliferate within various host cells. However, a recent study revealed that inhibiting glycolysis with 2-deoxy-D-glucose had no impact on *T. gondii* infection efficiency, suggesting that increasing glycolysis may be irrelevant for the control of *T. gondii*. Instead, mitochondrial OXPHOS may rather be relatively more important (67). Hence, from the perspective of energy supply, the energy metabolic reprogramming of host cells maintains its own energy requirements. Glycolysis activation is more likely a compensatory metabolic pathway triggered by the host in response to *T. gondii* infection, particularly due to mitochondrial dysfunction, rather than direct manipulation by the parasite. The above discussion suggests that glycolysis activation is beneficial to the survival of both host cells and *T. gondii*. However, the indisputable fact is that glycolysis activation results from mitochondrial dysfunction caused by *T. gondii* infection, which disrupts the cellular OXPHOS process. Recent studies have shown that aerobic glycolysis and OXPHOS are not mutually exclusive, with flexible metabolic phenotypes that shift in response to the specific conditions within the microenvironment (68). These dual interactions allow *T. gondii* to rapidly respond to changes in nutrient availability to maximize cell proliferation and survival.

In this study, quantitative indicators, including medium acidification, glucose consumption, and lactate and pyruvate productions, confirmed that *T. gondii* infection increased glycolytic metabolic activity in PK-15 cells. In addition, this study provided compelling evidence for the first time, from a molecular perspective, that *T. gondii* infection induced host cell metabolic reprogramming, along with key targets involved, including glycolysis-related enzymes PKM2, GLUT1, PDK1, PFKFB2, HK2, and LDHA, which were significantly upregulated in the infected cells. Previous research has shown that GLUT1, with high affinity for glucose, is responsible for the transport of glucose across the cell membrane and plays a crucial role in glucose uptake, which is considered a prerequisite for the enhanced glycolytic metabolism in tumor cells (69). The three rate-limiting enzymes HK2, PFKFB2, and PKM2 together control glycolytic flux (70); activation of PDK1 reduces mitochondrial OXPHOS and shifts cellular metabolism toward glycolysis (71). These targets may play decisive roles in *T. gondii*-induced glycolytic activation. Their abnormal expressions alter glycolytic activity and energy levels in cells and may have significant impacts on parasite survival. Nonetheless, one of the important issues that still needs to be addressed is how *T. gondii* causes mitochondrial dysfunction in host cells and activates glycolysis.

**Fig 10:**
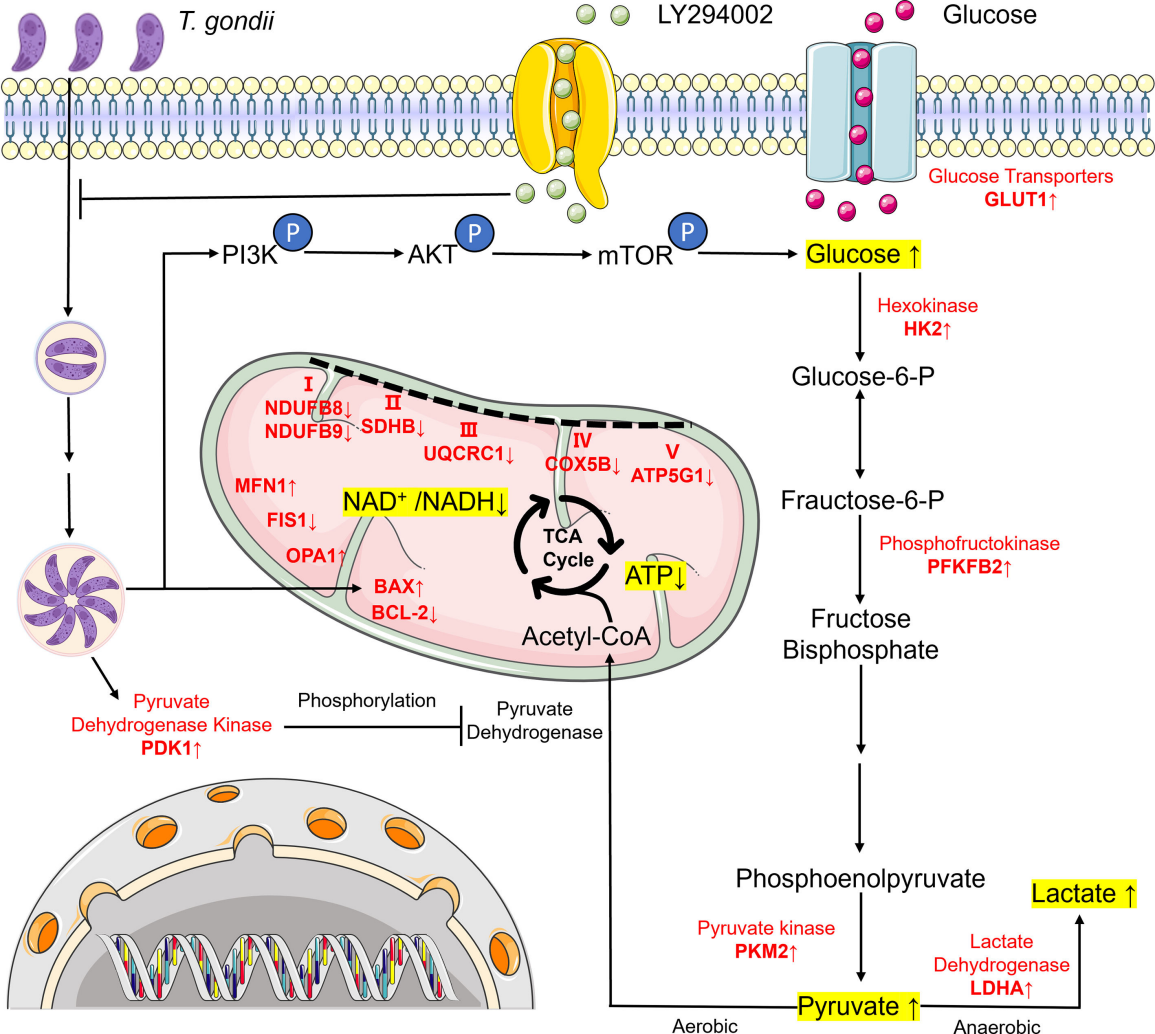
Schematic representation of how *T. gondii* induces the activation of the PI3K-Akt-mTOR pathway to regulate metabolic reprogramming and mitochondrial homeostasis in PK-15 cells.

The PI3K signaling pathway is a critical regulator of glycolysis. Akt, a downstream effector of PI3K, serves as a key driver of glycolytic phenotypes in tumors, enabling cancer cells to rely on glycolysis for survival. Akt is often referred to as the “Warburg kinase” (72). The activated PI3K/Akt pathway acts on the downstream molecule mTOR to regulate the expression of glycolysis-related enzymes and promote the Warburg effect (73). Under normal conditions, mTOR enhances PKM2 expression, facilitating tumor progression (74, 75). The PI3K/Akt pathway has also been reported to regulate GLUT1 expression (76, 77). Akt stimulates glucose uptake and glycolysis by increasing the expression and membrane translocation of glucose transporters such as GLUT1. Additionally, Akt modulates the expression and activity of HK2 and interacts with mitochondria to further amplify glucose uptake and glycolysis (78). In breast, lung, and pancreatic cancers, increased GLUT1 expression during carcinogenesis has been observed, leading to heightened glucose uptake by tumor cells (79), thereby disrupting glucose metabolism (80). Furthermore, PDK1 has been shown to activate the PI3K/Akt pathway via Akt, promoting glycolysis in cancer cells (81). The results of this study support these views. Consistent with the regulatory mechanism of tumor metabolism, *T. gondii* infection mediates glycolysis activation through the PI3K/Akt/mTOR signaling pathway (82–84), and meanwhile further proves that PKM2, GLUT1, PDK1, and HK2 are important targets regulating glycolysis via the PI3K/Akt/mTOR signaling pathway. Furthermore, recent studies have revealed that the PI3K/Akt signaling pathway also plays a significant role in regulating mitochondrial function, particularly mitochondrial metabolism. Despite being at a preliminary stage, Akt has been indicated to exert multiple activities in controlling mitochondrial metabolism, affecting both the TCA cycle and OXPHOS (85–87). Active Akt significantly enhances ATP production by modulating the expression of respiratory chain complexes I, III, and IV (87). Additionally, the PI3K/Akt/mTOR signaling pathway has been shown to influence mitochondrial dynamics, with its activation promoting mitochondrial fusion in cells (88), which is consistent with this study. A specific inhibitor, LY294002, that blocks the PI3K/Akt pathway, was used to block *T. gondii*-induced Akt phosphorylation in this study and was found to restore mitochondrial function (89). Inhibition of the PI3K/Akt/mTOR pathway significantly ameliorated *T. gondii*-induced alterations in mitochondrial morphology, dynamics, and membrane potential levels. More importantly, suppressing PI3K/Akt/mTOR activation demonstrated a robust restorative effect on the metabolic shift from OXPHOS to glycolysis induced by *T. gondii*. These findings further support the involvement of the PI3K/Akt/mTOR signaling pathway in regulating *T. gondii*-induced mitochondrial dysfunction and metabolic reprogramming. Combined with the above findings, we propose a model for the molecular mechanism by which *T. gondii* induces mitochondrial dysfunction and metabolic reprogramming (Fig. 10). Although LY294002 exhibited promising anti-*T*. *gondii* activity *in vitro*, all current findings are derived from cell culture systems. Therefore, future studies should evaluate its efficacy and safety *in vivo*. However, since LY294002 is a broad-spectrum PI3K inhibitor, its systemic application may lead to off-target toxicity (90). To address this, subsequent research should explore tissue-specific delivery strategies (91) or utilize more selective inhibitors, such as rapamycin, which targets downstream effectors of PI3K (92). Collectively, these findings provide critical insights into the mechanisms underlying *T. gondii*-induced metabolic reprogramming in host cells and lay a theoretical foundation for the development of novel therapeutic strategies.

## Data Availability

The authors confirm that the data supporting the findings of this study are available within the article.
